# The growth and developmental of the myodural bridge and its associated structures in the human fetus

**DOI:** 10.1038/s41598-023-40709-1

**Published:** 2023-08-17

**Authors:** Yang Song, Hua-Xun Lai, Ting-Wei Song, Jin Gong, Bo Liu, Yan-Yan Chi, Chen Yue, Jing Zhang, Shi-Zhu Sun, Cheng-Hong Zhang, Wei Tang, Ning Fan, Wei-Hua Yu, Yi-Fei Wang, Gary D. Hack, Sheng-Bo Yu, Jian-Fei Zhang, Hong-Jin Sui

**Affiliations:** 1 Department of Anatomy, College of Basic Medicine, Dalian Medical University, Dalian, 116044 Liaoning China; 2 Department of Neurology, Angang Group Company General Hospital, Anshan, 114000 Liaoning China; 3 Department of Histology and Embryology, College of Basic Medicine, Dalian Medical University, Dalian, 116044 Liaoning China; 4 Basic and Clinical Medicine Teaching Laboratory, School of Medicine, Sun-Yat-Sen-University, Guangdong, 518100 China; 5 Morphology Laboratory, College of Basic Medicine, Dalian Medical University, Dalian, 116044 Liaoning China; 6 Department of Obstetrics and Gynecology, Lvshun District Hospital, Dalian, 116044 Liaoning China; 7 Department of Obstetrics and Gynecology, Affiliated Zhongshan Hospital of Dalian University, Dalian, 116001 Liaoning China; 8 Department of Advanced Oral Sciences and Therapeutics, University of Maryland School of Dentistry, Baltimore, MD 21201 USA

**Keywords:** Developmental biology, Embryology, Morphogenesis

## Abstract

Myodural bridge (MDB) is a dense connective tissue between suboccipital muscle and dura mater. However, there are few reports on the development and maturation of the human MDB. This study aims to explore the developmental relationship between suboccipital muscle and MDB. 30 head and neck specimens from human fetuses (F) ranging from the 12th to 41st week (W) were made into histological sections. The F12W sections showed evidence that the dura mater dominated by fibroblasts, attached to the posterior atlanto-axial membrane (PAAM) which completely sealed the atlanto-axial space. In the F13W stage, myofibrils of the suboccipital muscle fibers increased significantly in number. At the F14W stage, a gap was observed at the caudal end of the PAAM. Numerous myodural bridge-like structures were observed blending into the dura mater through the gap. At the F19W stage, muscle cells mature. Starting at the F21W stage, the MDB were observed as fibroblasts that cross the atlanto-axial interspace and attach to the dura mater. Therefore, the traction generated by the suboccipital muscles seems to promote the maturity of MDB. This study will provide new morphological knowledge to support future research on the function of the human MDB and regulating the development mechanism of MDB.

## Introduction

In 1995, Hack et al. introduced the term myodural bridge (MDB)^1^. The MDB is a dense fibrous structure passing through both the posterior atlanto-occipital interspace^2^ as well as the posterior atlanto-axial interspace^3^. The MDB connects several of the suboccipital muscles and ligaments to the spinal dura mater. The relevant suboccipital muscles are the rectus capitis posterior minor (RCPmi), the rectus capitis posterior major (RCPma), and the obliquus capitis inferior (OCI)^4,5^, and the ligaments include the ligamentum nuchae (LN)^6,7^, the vertebral dura ligament (VDL) and the to be named ligament (TBNL)^8^. Recently, numerous papers have described the MDB in humans. Kitamura et al. described the candidate myodural bridge structure in the suboccipital region of human fetuses, and these fascial structures have many origins^9^. Zheng et al. found that the MDB of humans is primarily formed by type I collagen fibers, which are arranged in parallel^10^, and put forward the concept of the ‘Myodural Bridge Complex’ (MDBC)^11^. Jiang et al. described the ultra-microstructure of the human MDB, which merged into the spinal dura mater and became part of the dura mater^12^.

Various researchers have suggested that the MDB has significant physiological functions. For example, the MDB was described to be associated with the transmission of proprioception^13,14^, to keep the subarachnoid space and the posterior cerebellomedullary cistern patent during head movements^15^. Yuan et al. suggested that the myodural bridge may be related to cervicogenic headache^16^. Moreover, the MDB has been described as one of the factors which may potentially affect the dynamic circulation of cerebrospinal fluid (CSF)^8,17^. Recent research found that suboccipital muscle hyperplasia results in a significant increase in intracranial pressure, and it can be reduced by severing MDB^18^.

However, to date there have been only a few limited studies on the developmental morphology of the human MDB, thus, the relationship between the development of the MDB and the suboccipital musculature remains unclear. In the present study, histological sections with hematoxylin–eosin staining were used to describe the development of the MDB and its muscular associations in the human fetus. The present study adds to the knowledge base of developmental morphology research of the human embryonic MDB and laid a morphological foundation for further exploring its potential function and regulating the development mechanism of MDB.

## Materials and methods

The present study was approved by the Biomedical Ethics Committee of Dalian Medical University (2022-004). Written informed consent was obtained from the fetal parents involved in the present study in accordance with the regulations of the ethics committee.

In this study, 30 human fetal specimens with the gestational age of 12th–41st weeks were sectioned and studied, the crown-rump length (CRL) was 60–365 mm long. The specimens were donated by the Department of Obstetrics and Gynecology at Lvshun District Hospital.

### Specimen preparation

The fresh fetal specimens were perfused with 10% formalin through the umbilical vein. After umbilical vein perfusion, the skin in the suboccipital region was removed and the specimens were fixed using 10% formalin. After the specimens were fully fixed, the smaller specimens (smaller than 15 W) were severed at the occipital protuberance and the upper edge of the shoulder respectively in the cases of the specimens. And for the specimens with large gestational age (greater than 15 W), the occipital bone was cut transversely at the occipital protuberance. Thereafter, the tissues were cut along both sides of the spine, the vertebra was severed at the fourth cervical vertebra. Finally, the suboccipital region of the fetuses, including occipital bone, atlas, axis, suboccipital muscles, spinal dura mater, and the spinal cord were removed along the anterior intervertebral space.

### Histological slices and H&E staining

The samples were decalcified using Jiang Weizhong Decalcifying solution (80 ml of hydrochloric acid, 70 ml of formic acid, 50 g of calcium trichloride, 25 ml of glacial acetic acid, 100 ml of formaldehyde and 900 ml of normal saline. This solution decalcifies quickly, has little influence on tissues and has good dyeing effect) until the bones in the tissues could be pierced easily by a needle. After the decalcification procedure, the samples were washed with flowing water for 12 up to 24 h. Regular dehydration, as well as transparency and paraffin embedding methods were utilized. The embedded tissue blocks were sectioned into 10 μm thick slices using a rotary microtome (Leica Micro HM450; Leica Microsystems GmbH, Wetzlar, Germany). For analysis with the light microscope, the sections were stained with hematoxylin eos in stain^11^ (provided by Shanghai yuanye Bio-Technology Co., Ltd.). To eliminate the observer's prejudice, we standardize the observation indicators (Fibroblasts, large cells with many processes; the nucleus is large, oblate, lightly colored, with obvious nucleoli; the cytoplasm is weakly basophilic, and the outline of cells on HE stained specimens is unclear. It develops into a fibrous cell, with small cells and few protrusions, showing a slender spindle shape; the nucleus is small, light in color and the nucleoli is not obvious; cytoplasmic weakly eosinophilic).

## Results

### Histological study of the fetus in the 12 to 13-week of gestation (Fig. 1a,b)

**Figure 1:**
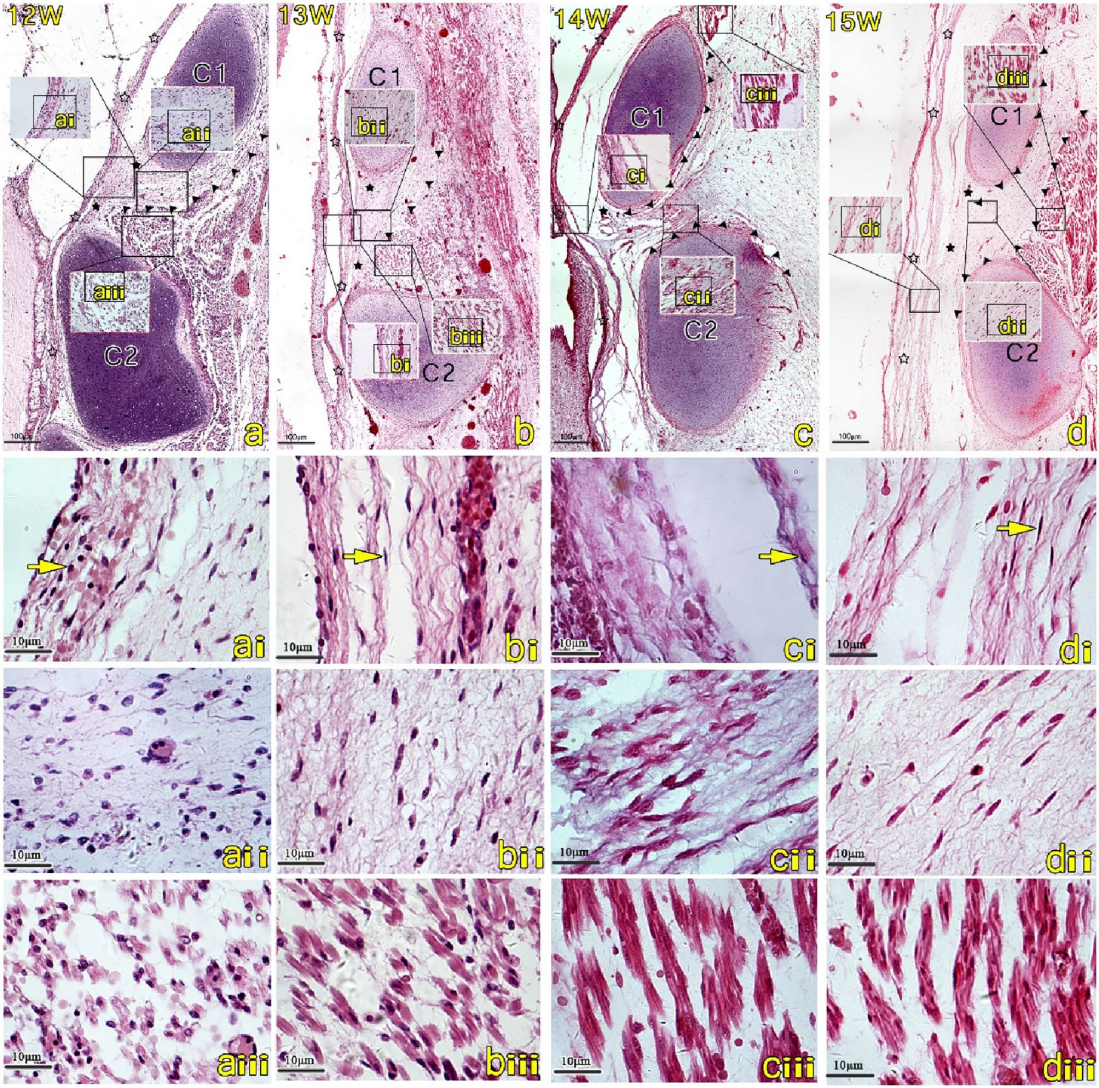
The posterior atlanto-axial interspace of the sagittal sections stained with HE stains at the 12 to 15-week human fetuses. The scale bars in images (**a**–**d**) are of the same 4 × magnification, while images (**a**–**d**(**i**–**iii**)) are of 40 × magnification. Images (**a**–**d**(**i**–**iii**)) are high-magnification views of the squares seen in images (**a**–**d**). The multi-layered dura mater (hollow star) closely attaches to the posterior atlanto-axial membrane (filled star) (**a**, **b**). The dura thickened gradually over time and transformed from fibroblasts (**ai**) to fibrocytes (**bi**–**di**) (yellow arrow). A gap appeared at the caudal end of the posterior atlanto-axial membrane and superior to the axis (C2), many noticeable myodural bridge-like structures (MDB) (filled arrowhead) blended into the dura mater through the gap (**c**, **d**). The orientation of cells of the myodural bridge fibers are now parallel and more orderly arranged (**aii**–**dii**). At this stage of development, the myofibrils of the suboccipital muscles gradually increased and the number of nuclei decreased (**aiii**–**diii**). *C1* atlas, *C2* axis, *MDB* myodural bridge.

Examining the sagittal sections obtained from the suboccipital region of 12-week gestation fetuses, the dura mater appeared to be thin and composed of multiple layers of thin and wavy fiber bundles. At this stage of development, there appeared to be primarily fibroblasts, each containing a large nucleus as well as obvious nucleoli in the spinal dura mater (Fig. 1ai). The connective tissue composing both the MDB and the posterior atlanto-axial membrane (PAAM) appeared to be relatively loose, and primarily composed of fibroblasts. We observed numerous short fiber bundles in the PAAM that appeared disordered with no obvious directionality (Fig. 1aii). From the 12th week onward specimens, muscle tissue was readily observed. However, the muscle fibers appeared to be relatively fewer in number and loose and were primarily in the form of myotubes or developing skeletal muscle fibers with a tubular appearance. Moreover, at this stage of development we observed that there were numerous nuclei and some myofibrils beginning to be arranged in parallel (Fig. 1aiii).

In the 13-week specimens, both the quantity of connective tissue in the suboccipital region and the thickness of the dura mater increased. Additionally, at this stage of development, the fiber bundles appeared to be thicker, and the cells contained within them were mainly fibrocytes. These fiber bundles appeared to run in a wave-like form (Fig. 1bi). Moreover, the nucleus appeared to be small and deeply stained, and the nucleoli were unclear. In comparison to the 12-week specimens, the fibers of the MDB and PAAM in the 13-week specimens appeared to be denser. Although these cells were observed to be primarily fibroblasts, the number of wavy fiber bundles increased, and their arrangement became more orderly (Fig. 1bii). Within the suboccipital muscles, the myofibrils became more numerous and denser when compared to the 12-week specimens, and numerous nuclei were still observed (Fig. 1biii).

### Histological study of the fetus in the 14 to 15-week of gestation (Fig. 1c,d)

In the study, it was observed from the 14th week onward specimens, the spinal dura gradually separates from the posterior wall of the spinal canal, and an epidural space appears (Fig. 1c,d). The dura mater is now very dense, and includes many typical fibrocytes with small deeply stained nuclei. There also appeared inconspicuous nucleoli and varying thicknesses of the fiber bundles which were oriented in a wavy shape (Fig. 1ci). At this stage, the MDB fibers are very thin and connected to the dura mater through the epidural space. The fibers of the MDB and PAAM have now lined up in parallel. Within the fibers, the fibroblasts still dominated (Fig. 1cii). Considering the suboccipital muscles, no obvious change in the number of myofibrils was observed (Fig. 1ciii,diii). At the F15W stage, the MDB fibers originated between the posterior arch of atlas (C1) and the obliquus capitis inferior muscle, and extended forward and downward into the spinal canal through the atlanto-axial gap.

### Histological study of the fetus in the 19-week of gestation (Fig. 2a)

**Figure 2:**
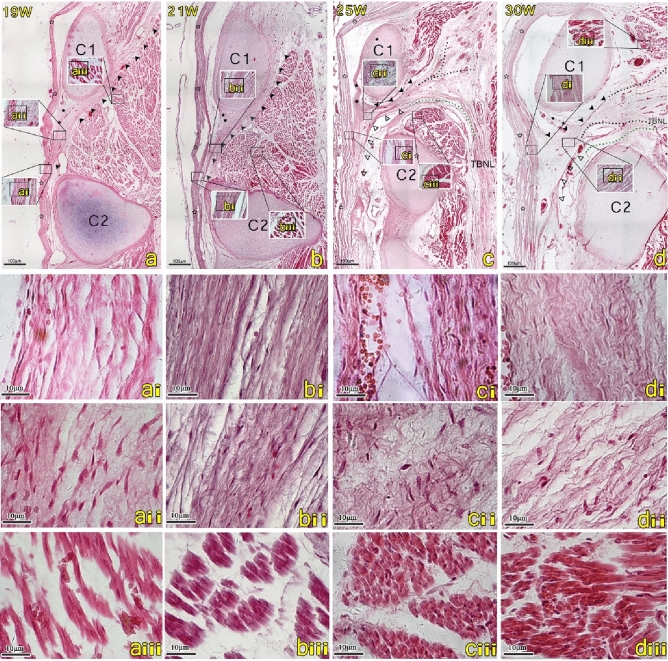
The H&E-stained sagittal sections from the posterior atlanto-axial interspace in human fetuses in the 19, 21, 25, and 30-week. The scale bars in images (**a**–**d**) are of the same 4 × magnification, while images (**a**–**d**(**i**–**iii**)) are of 40 × magnification. Images (**a**–**d**(**i**–**iii**)) are high-magnification views of the squares seen in images (**a**–**d**). The fibers of the MDB (filled arrowhead) pass through the posterior atlanto-axial membrane (PAAM) (filled star) within the posterior atlanto-axial interspace (**a**–**d**). (**a**, **b**) Fibers (filled arrowhead) travel longitudinally through the atlanto-axial interspace and extend downwards and forwards, eventually fusing with the dura mater (hollow star). (**c**, **d**) Most of the dense fibers (filled arrowhead) originate from the suboccipital muscles, and a few fibers arise from the TBNL (hollow triangle). The fibers of the dura mater are now significantly denser (**ai**–**di**). The MDB cells are mainly fibroblasts (**aii**) and gradually become fibrocytes (**bii**). Fiber bundles tend to be oriented parallel to each other (**bii**–**dii**). The morphology of the muscles is relatively mature (**aiii**–**diii**). *C1* atlas, *C2* axis, *TBNL* To Be Named Ligament, *Black dotted line* direction of the fibers arising from the posterior occipital muscles, *Green dotted line* direction of the fibers arising from the TBNL.

In the 19-week, the thickness of the dura mater continued to increase (Fig. 2ai). However, the fibers in the posterior atlanto-axial interspace now exhibited a unique shape. Although it is connected to the dura mater, the fibers of the PAAM do not extend from the muscle to the dura mater and run longitudinally between C1 and C2. The cells in the MDB and PAAM are still dominated by fibroblasts (Fig. 2aii). At this stage, the cells nuclei of the suboccipital muscles have now been deflected to one side, and the myotubes are filled with myofibrils. (Fig. 2aiii).

### Histological study of the fetus from the 21 to 30-week of gestation (Fig. 2b–d)

After 21 weeks, the morphological characteristics of the suboccipital region have stabilized. The fibers of the dura mater, MDB, and PAAM have now all become denser. The cells within these structures have now become fibrocytes, and their fiber bundles are arranged in parallel. The suboccipital muscles have also become loaded (Fig. 2b–d). Moreover, after observing the suboccipital muscles in different sections, it can be observed that the dense MDB fibers have the potential to pull the dura mater away from the spinal cord. In the section showing the To Be Named Ligament (TBNL), the posterior atlanto-axial interspace is now almost completely open with only a few fibers, and the dura mater is hardly stretched (Fig. 2c).

### Histological study of the fetuses after 31 weeks of gestation (Fig. 3a–d)

**Figure 3:**
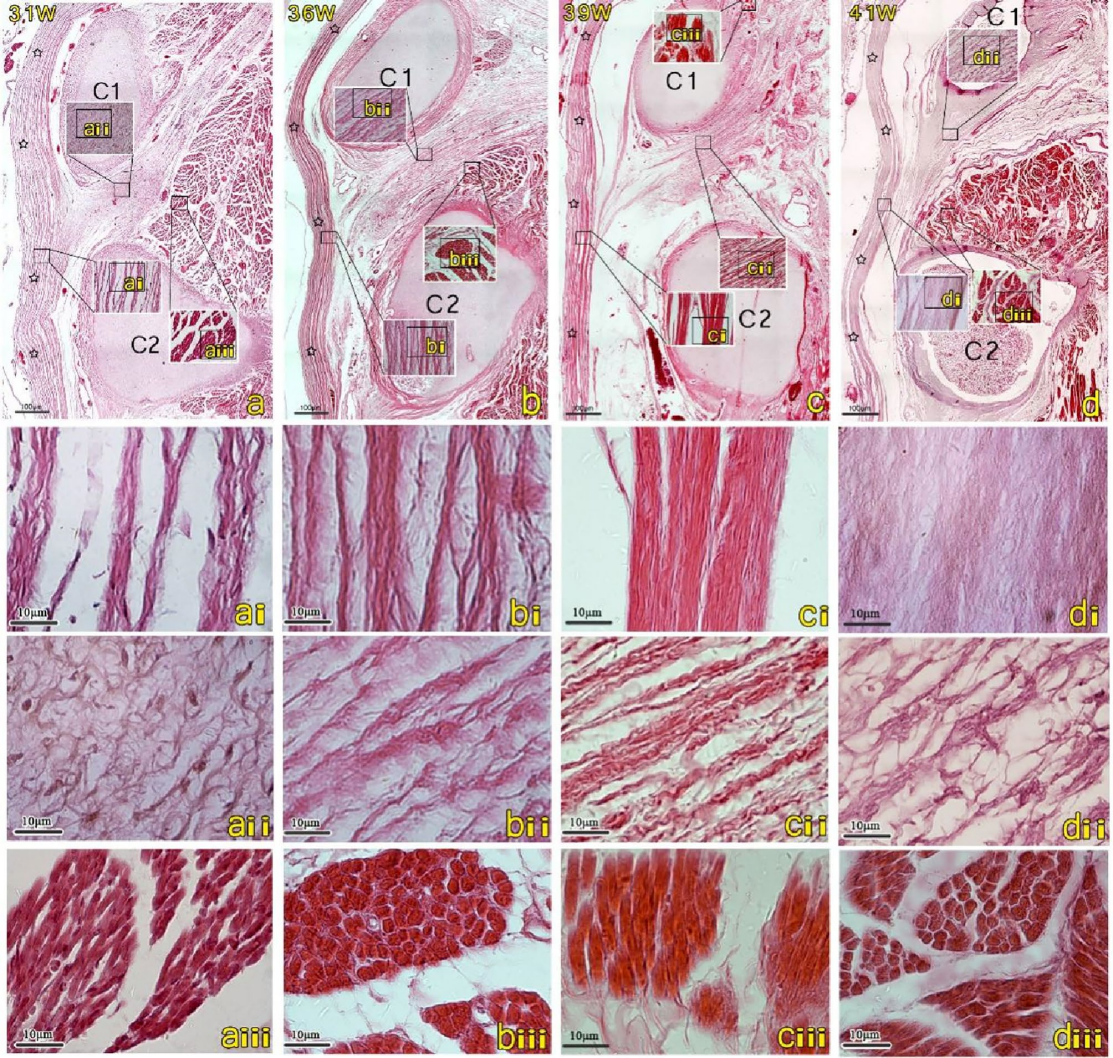
The H&E-stained sagittal sections of the posterior atlanto-axial interspace in the 31, 36, 39, and 41-week human fetuses. The scale bars in images (**a**–**d**) are of the same 4 × magnification, while images (**a**–**d**(**i**–**iii**)) are of 40 × magnification. Images (**a**–**d**(**i**–**iii**)) are high-magnification views of the squares seen in images (**a**–**d**). The cells of the dura mater and MDB have essentially become fibroblasts, the fibers have become thicker and denser (**ai**–**di**, **aii**–**dii**). Muscle fibers have become more mature and loaded (**aiii**–**diii**). *C1* atlas, *C2* axis.

After 31 weeks, the development of the suboccipital region has stabilized and the cellular morphology is mature. The cells of the dura mater and MDB have now differentiated into fibrocytes. As the fetus develops, the fibers become thicker and denser. The suboccipital muscles have matured, as well. Each muscle fiber has become fuller, and the intercellular space between muscle fibers has become smaller.

## Discussion

The human MDB was first reported in 1995^1^. Over the past 20 years, numerous researchers have explored its function^8,13–15,17,18^, and studies on the MDB have included anatomy, histology, and clinical pathology. The most important function is its influence on cerebrospinal fluid circulation^8,17^. Moreover, some scholars suggest that severing MDB can effectively reduce increased intracranial pressure caused by suboccipital muscle hyperplasia, and provide a unique therapeutic prospective for physicians relating to unexplained long-term headaches^18^. However, few studies have been done on the development and maturation of the MDB in human fetuses, which limits the further detailed study on MDB. To fully understand the structure and function of the MDB, it is necessary to explore the growth and development of the MDB and its related structures (the spinal dura mater and the suboccipital musculature).

Some studies have described that the cervical region develops from 2, 3, 4 and 6 pairs of branchial arches: at the 4th–5th week, the 2nd branchial arch rapidly grows caudally. The 2nd–4th branchial sulcus form a cervical sinus in the deep part of the 2nd branchial arch. With the differentiation of branchial arch, the neck is gradually lengthened and shaped^19,20^. The description of this part of early embryo development can help us to locate when preparing small gestational age embryo samples.

Studies have verified that the dura mater becomes apparent in the 8–9 week^21^. The present study, demonstrates that in the12-week, the dura mater can be readily recognized and presents as a multilayer fibrous structure. At this stage of development, the dura mater appears to be relatively thin, and its constituent cells are primarily fibroblasts. In the 13-week of development, the dura mater appears thicker, and its cells are now mainly fibrocytes. From the 12th to 13th week of maturation, the dura mater appears attached to the posterior wall of the spinal canal, and to the PAAM. At the 14-week stage, the dura mater becomes detached from the posterior wall of the spinal canal with some fibrous formations connecting them within the posterior atlanto-axial interspace. Researchers suggest that active fetal movement is the cause of the secondary epidural space, with the lateral and posterior portions of the spinal dura being separated from the spinal canal^21^. During subsequent development, the dura mater gradually thickens and becomes denser, and the fibers connecting the dura mater to the posterior wall of the spinal canal also become denser. The atlanto-occipital and atlanto-axial interspaces have a unique tissue morphology, and there are dense fibrous connections between suboccipital muscles and dura mater. These fibrous formations have been referred to as the MDB in adults^1^.

At the 12-week stage, myotubes can be readily observed in the suboccipital muscles (Fig. 1aiii) with mature muscular ducts appearing^22^. During subsequent development, the myotubes undergo further cell fusion. Additionally, during this stage, there appears the expression of contractile proteins forming mature muscle fibers and developing into functioning muscles with contractile capabilities^23^. After 12 weeks, the myotubes appear to have differentiated into muscle fibers, representing the final stage in the differentiation of skeletal muscle. Through our morphological observations, the morphology of the muscles appears mature in the 19-week (Fig. 2aiii).

**Figure 4:**
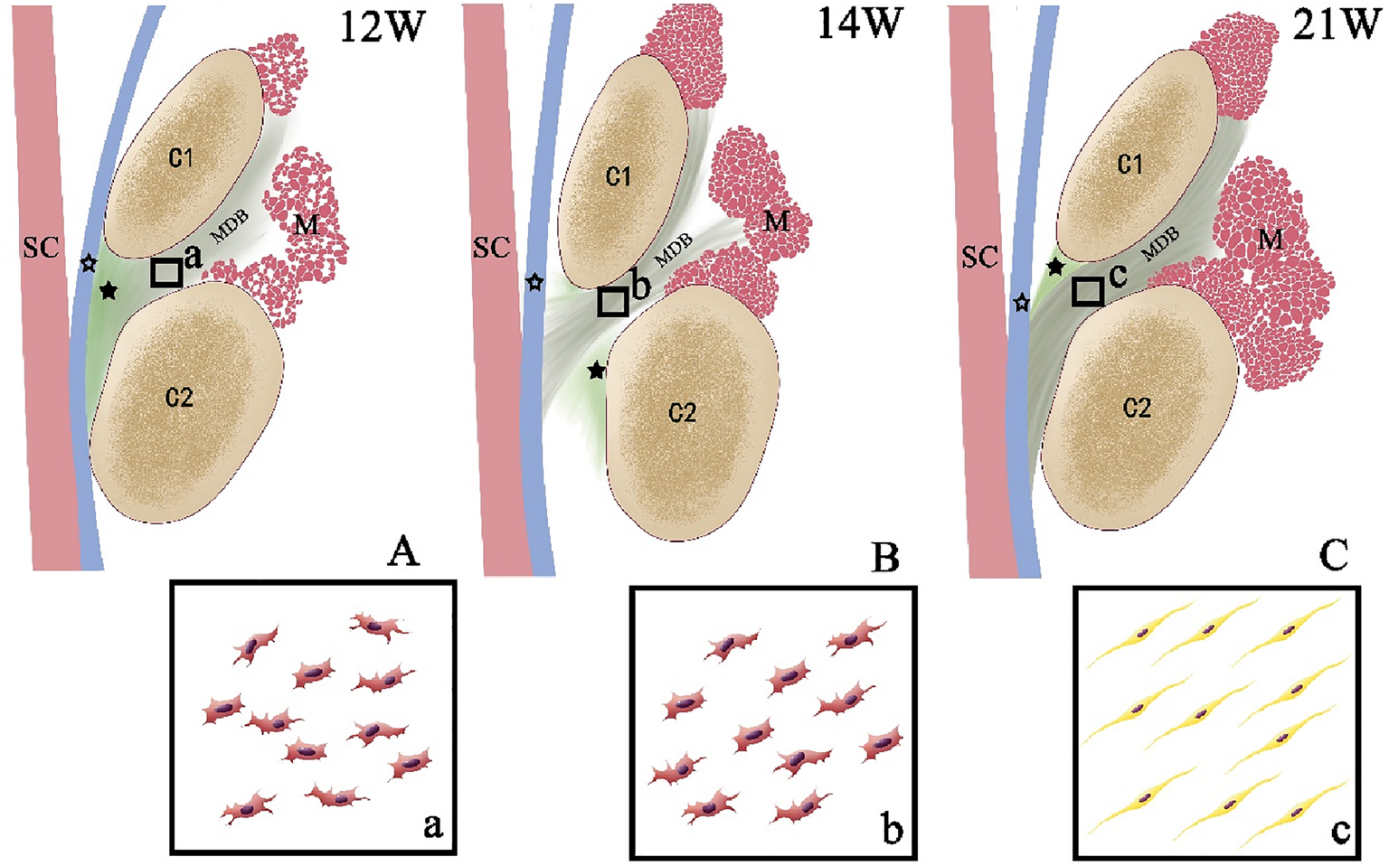
The posterior atlanto-axial interspace of the mode diagram in the 12, 14 and 21-week human fetuses. Images a-c are high-magnification views of the squares seen in images (**A**–**C**). The dura mater (hollow star) closely attaches to the posterior atlanto-axial membrane (filled star) (**A**). Myodural bridge-like structures (MDB) are mainly disordered fibroblasts in the 12-week (a). A gap appeared in the posterior atlanto-axial membrane above the axis (C2), and many MDB connected to the dura mater through the gap (**B**), and the orientation of fibroblasts of the MDB are now parallel and more orderly arranged (b). The morphology of the muscles is relatively mature from19 week (**C**). Muscle became more mature from 19 weeks, and MDB became orderly fibrocytes in the 21-week (c). *C1* atlas, *C2* axis, *MDB* myodural bridge, *SC* spinal cord, *M* the posterior occipital muscles.

The MDB fibers are already in place in the 12-week and continue to differentiate progressively within the connective tissue (Fig. 4A,a). In the 12 to 13-week, the cells constituting the MDB are primarily fibroblasts, but the fibers themselves are disorderly arranged. From the 14th week onward specimens, although the cells of the MDB remain primarily fibroblasts, the arrangement of the fibers has now become more orderly and arranged in a parallel order (Fig. 4B,b). In the 14-week specimen, it can be observed that the dura is separated from the posterior wall of the spinal canal. Interestingly, it is known that fibroblasts form linear bundles along the direction of traction during development^24^. Therefore, the separation of the dura mater from the posterior wall of the spinal canal may provide tractional forces from the MDB and cause the fiber arrangement in the dura to become more orderly. In the 21-week specimen, the fibers of the MDB appear denser, and the cellular components of the MDB are now dominated by fibrocytes (Fig. 4C,c). Previous studies have shown that tension generated by the RCPmi muscle can cause the dura mater to move via the transmission of forces from the MDB^25^. The present study found that the MDB matures later than the suboccipital musculature. From the 25th week to the 30th week, some of the MDB fibers were observed to be originating from the To Be Named Ligament (TBNL), which is a dense fibrous structure emanating from the posterior part of the nuchal ligament (NL)^8^. It was also observed that the MDB at the suboccipital muscle level is denser and more orderly than the MDB located at the TBNL level. Therefore, our results show that the MDB is stretched by the suboccipital musculature during its development.

Previous studies have suggested that when the dura mater bridge plays the role of transmitting force, it may be closely related to the contraction of suboccipital muscles^26^. Muscle development is also closely related to fetal activity: mothers can usually begin to feel their babies movements in their uterus from around the 16th to 20th week^27^. In fact, fetal movement happens before the mother detects them because the movements are felt by the mother only when they are sufficiently strong enough to stimulate the abdominal wall^28^. Some scholars showed that head movement was helpful to promote the circulation of cerebrospinal fluid (CSF), and MDB may transform tensional forces generated by the suboccipital muscles^17^. It has been reported that the fetus has obvious head movement in the 14 to 16-week of gestation^29^. This is probably related to the separation of the spinal dura from the spinal canal in the 14-week in the present study. According to Shiota^30^, two mechanical factors can mobilize the dura mater: differential growth between the spinal cord, the vertebral column, and the dura mater, and fetal movements. From the 17 to 19-week, the fetus’s head moves more frequently^29^ and the development of the suboccipital musculature is becomes fully mature, while the MDB becomes mature in the 21-week. Therefore, we believe that head movement and muscle activation help to promote the development of the MDB. This study has some limitations: the acquisition of embryos is random and the gestational age cannot be specified, and samples of smaller gestational age cannot be obtained. Based on the results of this study, the further researches about the genes and molecular mechanisms regulating myodural bridge development, and the evolution process of MDB from aquatic to terrestrial of vertebrates will be conducted. Theses researches would reveal the physiological mechanisms of the theory that MDB affects cerebrospinal fluid circulation, illuminate the pathophysiological processes of cerebellar tonsillar hernia and syringomyelia in clinic, and provide new ideas for the etiology analysis and treatment of related diseases^31^.

## Conclusion

The present study explored cell differentiation of the dura mater, the MDB, and the suboccipital musculature during the development and growth of the suboccipital region. The cells of the spinal dura mater matured in the 13-week, whereas muscle cells matured in the 19-week. The MDB cells showed the morphology of fibrocytes in the 21-week. Therefore, the tractional forces generated by the suboccipital muscles appears to promote the maturation of the MDB. The results of this study laid a foundation for further study on the biomechanics and function of MDB, and also laid a morphological foundation for the development of human embryos for the next step to explore the mechanism of regulating MDB.

## Data Availability

All data generated and analyzed during this study are included in this article. All the data was obtained via the above experiments.
